# Digitally Assisted Peer Recovery Coach to Facilitate Linkage to Outpatient Treatment Following Inpatient Alcohol Withdrawal Treatment: Proof-of-Concept Pilot Study

**DOI:** 10.2196/43304

**Published:** 2023-07-05

**Authors:** Joji Suzuki, Frank Loguidice, Sara Prostko, Veronica Szpak, Samata Sharma, Lisa Vercollone, Carol Garner, David Ahern

**Affiliations:** 1 Department of Psychiatry Brigham and Women's Hospital Boston, MA United States; 2 Harvard Medical School Boston, MA United States; 3 Department of Internal Medicine Brigham and Women's Faulkner Hospital Boston, MA United States

**Keywords:** alcohol use disorder, inpatient detoxification, peer recovery coach, smartphone app, alcohol, substance use, substance abuse, drinking, recovery, peer support, detox, coaching, health app, mobile health, mHealth, mobile app, care coordination, digitally, detoxification, Lifeguard, peer recovery, inpatient alcohol

## Abstract

**Background:**

Alcohol use disorder (AUD), associated with significant morbidity and mortality, continues to be a major public health problem. The COVID-19 pandemic exacerbated the impact of AUD, with a 25% increase in alcohol-related mortality from 2019 to 2020. Thus, innovative treatments for AUD are urgently needed. While inpatient alcohol withdrawal management (detoxification) is often an entry point for recovery, most do not successfully link to ongoing treatment. Transitions between inpatient and outpatient treatment pose many challenges to successful treatment continuation. Peer recovery coaches—individuals with the *lived experience* of recovery who obtain training to be coaches—are increasingly used to assist individuals with AUD and may provide a degree of continuity during this transition.

**Objective:**

We aimed to evaluate the feasibility of using an existing care coordination app (Lifeguard) to assist peer recovery coaches in supporting patients after discharge and facilitating linkage to care.

**Methods:**

This study was conducted on an American Society of Addiction Medicine–Level IV inpatient withdrawal management unit within an academic medical center in Boston, MA. After providing informed consent, participants were contacted by the coach through the app, and after discharge, received daily prompts to complete a modified version of the brief addiction monitor (BAM). The BAM inquired about alcohol use, risky, and protective factors. The coach sent daily motivational texts and appointment reminders and checked in if BAM responses were concerning. Postdischarge follow-up continued for 30 days. The following feasibility outcomes were evaluated: (1) proportion of participants engaging with the coach before discharge, (2) proportion of participants and the number of days engaging with the coach after discharge, (3) proportion of participants and the number of days responding to BAM prompts, and (4) proportion of participants successfully linking with addiction treatment by 30-day follow-up.

**Results:**

All 10 participants were men, averaged 50.5 years old, and were mostly White (n=6), non-Hispanic (n=9), and single (n=8). Overall, 8 participants successfully engaged with the coach prior to discharge. Following discharge, 6 participants continued to engage with the coach, doing so on an average of 5.3 days (SD 7.3, range 0-20 days); 5 participants responded to the BAM prompts during the follow-up, doing so on an average of 4.6 days (SD 6.9, range 0-21 days). Half (n=5) successfully linked with ongoing addiction treatment during the follow-up. The participants who engaged with the coach post discharge, compared to those who did not, were significantly more likely to link with treatment (83% vs 0%, *χ*^2^=6.67, *P*=.01).

**Conclusions:**

The results demonstrated that a digitally assisted peer recovery coach may be feasible in facilitating linkage to care following discharge from inpatient withdrawal management treatment. Further research is warranted to evaluate the potential role for peer recovery coaches in improving postdischarge outcomes.

**Trial Registration:**

ClinicalTrials.gov NCT05393544; https://www.clinicaltrials.gov/ct2/show/NCT05393544

## Introduction

Alcohol use disorder (AUD), associated with significant morbidity and mortality, continues to be a major public health problem. The COVID-19 pandemic exacerbated the impact of AUD, with a 25% increase in alcohol-related mortality from 2019 to 2020. As such, innovative treatments for AUD are urgently needed. Inpatient alcohol withdrawal management (ie, detoxification) accounts for over 20% of the annual admissions to publicly funded facilities to treat substance use disorders [1]. Such admissions are often an entry point to recovery for individuals with AUD, and a successful linkage to ongoing treatment has been shown to improve the outcomes [2]. The types of treatment to which patients are linked can vary considerably and may include additional intensive interventions such as partial hospital, residential, and intensive outpatient services, or a range of outpatient treatments such as outpatient counseling and attendance at 12-step programs. Although estimates vary, only 30%-35% of patients receive some form of addiction treatment after discharge [3]. One of the major barriers for successful linkage is the fragmentation of detoxification treatment from outpatient addiction treatment services [4].

Peer recovery coaches are deployed increasingly in health care settings, often providing services outside the scope of traditional clinical services [5]. Recovery coaches have the “lived experience” of sustained recovery who obtain training to become certified to provide support for individuals in recovery [6]. Recovery coaches’ roles vary tremendously depending on the setting and patient population, ranging from single encounters in the emergency department to provide referral to services, to providing ongoing support to patients longitudinally over time [7,8]. What is consistent across these various roles, however, is that peer recovery coaches often provide social support, build trust, respect all pathways to recovery, instill hope, and assist in navigating community recovery resources [6].

Additionally, mobile technologies are increasingly studied to improve treatment outcomes for a variety of illnesses, including AUD [9]. Prior trials that have combined smartphones with peer recovery coaches have included primary care patients with hazardous drinking [10,11] or individuals in the emergency department with opioid use disorder [12]. However, there are no prior studies that have attempted to leverage technology to increase engagement with peer recovery coaches to specifically improve linkage to care following inpatient alcohol withdrawal management treatment.

To fill this gap, we conducted a proof-of-concept study to evaluate the feasibility of an existing care coordination platform and smartphone app (Lifeguard) in facilitating recovery coach support for patients being discharged from detoxification. Lifeguard is a smartphone app that was designed to assist patients, their caregivers, and their providers by giving reminders about upcoming appointments and medications, allowing caregivers to remotely support the patient, tracking symptoms such as vital signs, giving providers patient-reported measures between visits, and creating care plans. We aimed to conduct a small proof-of-concept trial to assess the feasibility of a peer recovery coach using this smartphone app to provide the support to patients undergoing inpatient withdrawal treatment, with the goal of improving linkage to ongoing addiction treatment after discharge.

## Methods

### Setting

This study was conducted at a Level IV-D Medically Managed Intensive Inpatient withdrawal management (ie, detoxification) unit in Boston, MA. Level IV units are the highest level designed to offer intensive medical care for those with signs and symptoms of alcohol withdrawal sufficiently severe to require 24-7 monitoring, including cardiac monitoring. All participants received the hospital’s standard detoxification protocol, using both pharmacotherapy and intensive psychosocial support. Therapists conduct a comprehensive psychosocial evaluation and offer daily individual and group therapy covering topics such as goal setting, relapse prevention, coping strategies, stages of change, and mindfulness. Therapists also address aftercare planning with all patients. Recruitment and data collection occurred between November 2021 and April 2022. The trial was registered in ClinicalTrials.gov (NCT05393544).

### Ethical Considerations

This study was approved by the Mass General Brigham Institutional Review Board (protocol# 2021P000731). All participants invited to participate in the trial provided full written informed consent prior to any study procedures being conducted. All staff were trained in confidentiality and through data security procedures, with data deidentified and coded with unique ID numbers and securely stored. The key linking participants’ names and ID numbers was stored in a separate password-protected document in a password-protected computer. Access to data storage areas was restricted to authorized study personnel, and all analyses were conducted on deidentified data.

### Participants

Eligible participants were individuals hospitalized for inpatient alcohol withdrawal management and possessed a smartphone. Exclusion criteria were the inability to provide consent due to impaired mental status and the need for inpatient treatment for psychiatric disorders.

### App Onboarding

After obtaining informed consent, the study staff assisted the participants to download the Lifeguard app, create a log-in and password, and demonstrated the basic functions, including how to respond to prompts and how to send and receive text messages.

### Recovery Coach

**Figure 1:**
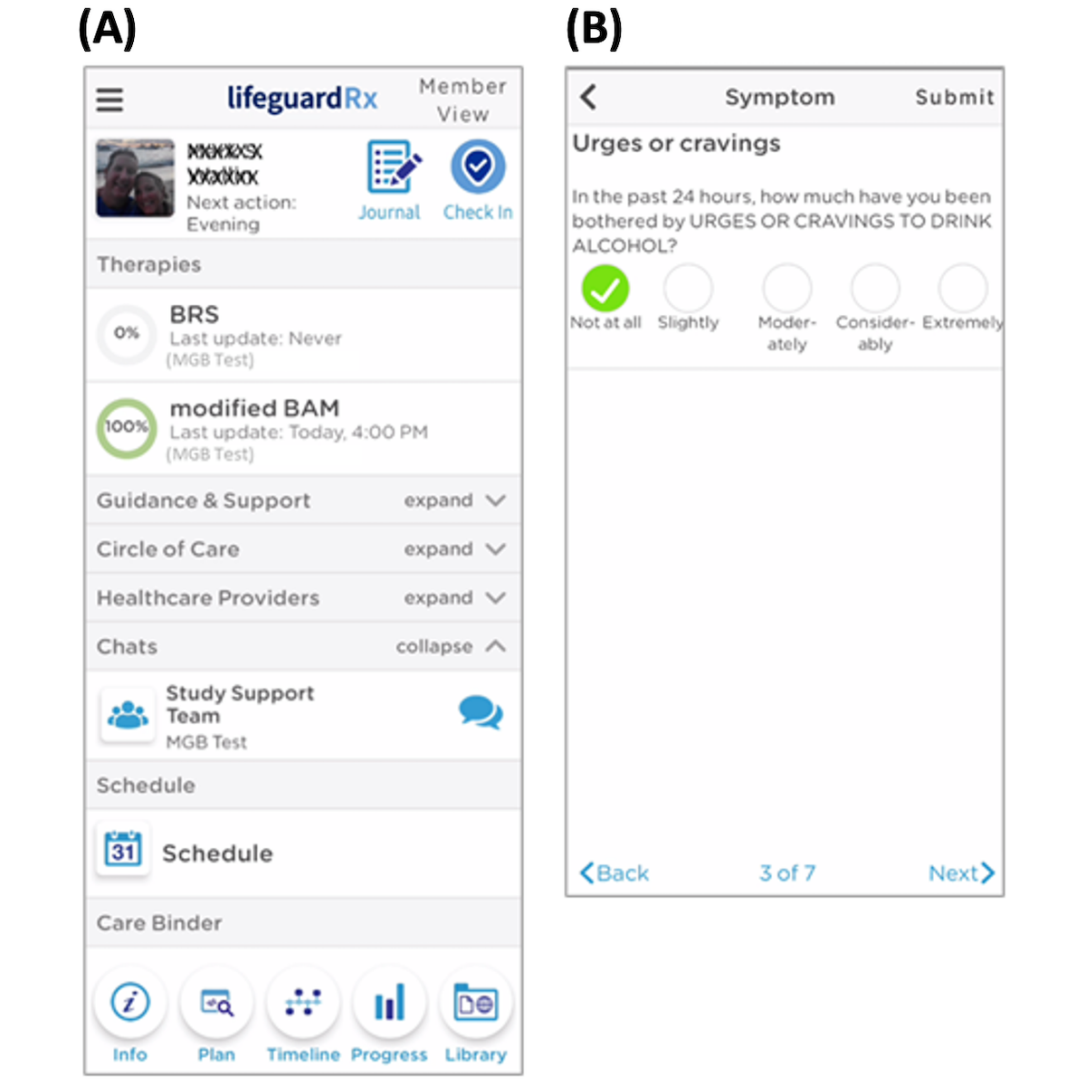
Sample screenshot of the Lifeguard app used for the trial. (A) Home screen with therapies and chat section exposed. (B) Example question from modified BAM assessment. BAM: brief addiction monitor; BRS: Buoyant Resilience Scale; MGB: Mass General Brigham.

The peer recovery coach (FL) engaged with the participant through the app prior to discharge to introduce himself and review how to use the app as well as his role in the intervention. The Lifeguard app was originally designed to facilitate care coordination between the patient, health care providers, and care givers, while also allowing for the monitoring of symptoms (Figure 1). For this study, the app was used by the recovery coach to facilitate contact with the participant and monitoring during the 30-day follow-up. The intervention consisted of the following features.

#### Motivational Messages

Text messages were sent each day at noon to participants, which were passages from the book of Daily Reflections [13]. The book, written by members of the Alcoholics Anonymous as a resource for those in recovery, is composed of short passages for each calendar day to be incorporated into a daily routine. These passages are about living sober from alcohol and are meant to focus on specific Alcoholics Anonymous principles. The participants were not expected to respond to these messages. Example text messages include, “One exercise that I practice that I try is for a full inventory of my blessings” and “The more we become willing to depend on a higher power, the more independent we become.”

#### Appointment Reminders

The coach sent reminder text messages for upcoming appointments for ongoing addiction treatment.

#### Brief Addiction Monitor

The Brief Addiction Monitor (BAM) [14] was modified for this study by selecting 7 questions that cover the 3 domains of “use” (alcohol use), “risk factors” (negative mood, cravings to drink alcohol, and being in risky situations), and “protective factors” (confidence in staying sober, attendance at self-help meetings, and being in contact with those supportive of their recovery). Participants received prompts to complete the BAM daily for 30 days after discharge.

#### Coach Check-in

If the BAM responses indicated alcohol use, the presence of risk factors, or not engaging in protective factors, then the coach checked in with the participant and referred the participant to the hospital’s bridge clinic for a clinical evaluation if needed.

### Compensation

We provided participants with a debit card that allowed funds to be added electronically as compensation. After enrollment, US $25 were provided to the participants while still on the inpatient unit, and the remaining US $25 were provided after the 30-day assessment was completed.

### Feasibility Outcomes

We examined the following outcomes to evaluate the feasibility: (1) the proportion of participants engaging with the coach before discharge, (2) the proportion of participants and the number of days engaging with the coach after discharge, (3) the proportion of participants and the number of days responding to BAM prompts, and (4) the proportion of participants successfully linking with addiction treatment by 30-day follow-up. A successful engagement for a particular day was defined as having any contact with the coach with a phone call or text message. All participants on the inpatient unit as part of routine clinical care were expected to create an aftercare plan regarding ongoing addiction treatment in the community, which typically involved either inpatient-level treatment (ie, residential) or outpatient services. Successful linkage to care was confirmed either via self-report of the participant or through the review of the chart if referral had been made to our own institution’s clinical program.

### Analytic Strategy

Descriptive statistics were used to summarize the data. Linkage to care was determined via self-report, and, where possible, confirmed through the electronic health record. A chi-square test of independence examined the relationship between engagement with the coach post discharge and successful linkage. Type I error was set at .05.

## Results

In total, we approached 24 individuals, of whom 12 were excluded for the following reasons: declined participation (n=3), discharged before enrollment could proceed (n=2), unable to contact participant for the screening visit (n=2), required inpatient psychiatric care for suicidal ideation (n=1), lacked capacity to provide consent (n=2), and did not have a phone (n=2). Of the 12 who provided informed consent, 2 were not enrolled due to technical difficulties with the app which the study staff could not resolve. A total of 10 individuals were found to be eligible and provided informed consent to participate in the study. The participants were all men with AUD admitted for inpatient withdrawal management; averaged 50.5 years old; and were mostly of White racial backgrounds (n=6), non-Hispanic (n=9), and single (n=8).

Given that this proof-of-concept study aimed to evaluate the feasibility, our outcomes of interest were primarily with the proportion of participants engaging with the coach before and after discharge, responding to BAM prompts, and successfully linking with addiction treatment by 30-day follow-up. Overall, 8 participants successfully engaged with the coach prior to discharge while still on the inpatient unit. Following discharge, 6 participants continued to engage with the coach, doing so on an average of 5.3 days (SD 7.3, range 0-20 days). During the 30 days post discharge, 5 participants responded to the BAM prompts, doing so on an average of 4.6 days (SD 6.9, range 0-21 days). Half of the participants were successfully linked with ongoing addiction treatment during the follow-up. The participants who engaged with the coach post discharge, compared to those who did not, were significantly more likely to link with treatment (83% vs 0%, *χ*^2^=6.67, *P*=.01). Responding to the BAM prompts was not associated with successful linkage (80% vs 20%, *χ*^2^=3.60, *P*=.06).

## Discussion

This proof-of-concept study evaluated the feasibility of a digitally assisted peer recovery coach in engaging patients undergoing inpatient alcohol withdrawal management. We additionally evaluated if coach engagement might facilitate linkage to ongoing treatment. While still preliminary, the results were promising in the feasibility outcomes examined, with most of the participants engaging with the coach and responding to the prompts during the follow-up period. The results also indicated that engagement with the coach was associated with a greater likelihood of linking to ongoing addiction treatment after discharge, suggesting possibly that the support provided by peers can positively impact postdischarge outcomes. Nevertheless, the total number of days that the participant engaged with the coach was limited, including the total number of days responding to BAM prompts, pointing to the challenges when in-person contact is absent. While in-person interventions are likely to have a greater magnitude of effect on the outcomes, there is a growing recognition for the role of digital technologies in improving clinical outcomes of individuals in recovery [15,16]. Taken together, the results suggest that further research is warranted to evaluate the benefits of recovery coaches to facilitate postdetoxification linkage to care through a smartphone app.

It remains unclear if providing support during the transition after inpatient treatment is the critical ingredient in enhancing continuing care for those in recovery. Prior trials to improve postdetoxification linkage to care have produced mixed results. In a randomized trial, a brief family treatment during the admission was superior to treatment as usual (TAU) in increasing entry to treatment at 30 days [17]. In another trial, a staff escort to the aftercare appointment along with a small incentive improved linkage with treatment compared to incentives alone or TAU [18]. In another trial that randomized participants to either a peer- or physician-led referral to 12-step programs or no active referral at all, those receiving a peer-led referral were significantly more likely to attend a 12-step meeting (64%) compared to physician-led referral (48%) or to controls (33%). Finally, patients receiving a visit from a nurse from the outpatient clinic to which patients were referred were significantly more likely to link with care compared with TAU [19]. By contrast, in a randomized trial comparing motivational enhancement therapy, peer-delivered 12-step facilitation, and TAU, linkage at 30 days was no different between the arms [20]. In another trial, patients receiving an enhanced telephone monitoring—consisting of a 50-minute in-person individual session and 12 weekly 15-minute phone calls after discharge—were no more likely to engage with outpatient treatment at 3-months compared to TAU [21]. These results suggest that more research is needed to identify how to improve postdetoxification linkage to care.

The BAM used for this trial was designed to deliver patient-reported measures to the peer recovery coach, who can then intervene if needed by providing support and referral to services. However, the overall response to BAM was limited (40% never responded), and of those who responded, the frequency of responding was low (mean of 4.6 out of 30 days). This may suggest that daily requests to complete the BAM may be too burdensome to participants. Nevertheless, the results suggested that those who responded to the BAM prompts may be more likely to engage with aftercare, although the results were not statistically significantly (*P*=.06). As such, further research is warranted to assess the optimal frequency for requesting patient-reported measures, as well as whether such prompts can enhance engagement with the peer recovery coach or improve linkage to care.

There are several limitations to this study. This was a small proof-of-concept pilot study, with no control group, and used a convenience sample at a single institution. Although we did not restrict enrollment by gender, only men chose to enroll. Most of the follow-up postdetoxification occurred at programs outside of our institution, which prevented confirmation of linkage via chart review. Finally, the lack of any in-person contact may have hampered the degree of engagement between the participant and the coach.

A recovery coach using a smartphone app may feasibly support patients following admission for inpatient withdrawal management. Further research is warranted to evaluate the potential role for peer recovery coaches in improving postdischarge linkage to treatment.

## Abbreviations

AUD: alcohol use disorder
BAM: brief addiction monitor
TAU: treatment as usual

## Data Availability

The data sets generated or analyzed during this study are available from the corresponding author on reasonable request.
